# Sodium and Lithium Storage Properties of Spray-Dried Molybdenum Disulfide-Graphene Hierarchical Microspheres

**DOI:** 10.1038/srep11989

**Published:** 2015-07-15

**Authors:** Sujith Kalluri, Kuok Hau Seng, Zaiping Guo, Aijun Du, Konstantin Konstantinov, Hua Kun Liu, Shi Xue Dou

**Affiliations:** 1 Institute for Superconducting and Electronic Materials, University of Wollongong, NSW 2500, Australia; 2 School of Mechanical, Materials and Mechatronics Engineering, University of Wollongong, NSW 2500, Australia; 3 School of Chemistry, Physics and Mechanical Engineering, Queensland University of Technology, Brisbane, Queensland 4001, Australia

## Abstract

Developing nano/micro-structures which can effectively upgrade the intriguing properties of electrode materials for energy storage devices is always a key research topic. Ultrathin nanosheets were proved to be one of the potential nanostructures due to their high specific surface area, good active contact areas and porous channels. Herein, we report a unique hierarchical micro-spherical morphology of well-stacked and completely miscible molybdenum disulfide (MoS_2_) nanosheets and graphene sheets, were successfully synthesized via a simple and industrial scale spray-drying technique to take the advantages of both MoS_2_ and graphene in terms of their high practical capacity values and high electronic conductivity, respectively. Computational studies were performed to understand the interfacial behaviour of MoS_2_ and graphene, which proves high stability of the composite with high interfacial binding energy (−2.02 eV) among them. Further, the lithium and sodium storage properties have been tested and reveal excellent cyclic stability over 250 and 500 cycles, respectively, with the highest initial capacity values of 1300 mAh g^−1^ and 640 mAh g^−1^ at 0.1 A g^−1^.

In recent years, room temperature sodium-ion batteries (SIBs) have been the object of significant interest for their potential application in large-scale energy storage systems. This is mainly caused by concerns about insufficient lithium ores to satisfy the increasing demands for lithium-ion batteries (LIBs), as well as because sodium is a relatively cheaper option compared to lithium, which could be significant in large-scale applications such as grid storage. In addition, the electrochemical principles of SIBs are identical to those of LIBs^1^. However, some anode materials which are suitable for LIBs may not be well compatible for SIBs. For instance, graphite, which is a commercial anode material in LIBs, delivers unsatisfactory electrochemical behavior in SIBs unless with specialized ether-based electrolyte solvents such as diethylene glycol dimethyl ether (DEGDME), tetraethylene glycol dimethyl ether (TEGDME) etc^2^,^3^. Thus it could be of research interest to explore the compatibility of various classes of anode materials for LIBs and SIBs.

Amongst various anode materials, molybdenum disulfide is one of the earliest compounds studied for rechargeable LIBs due to its layered structure, which can intercalate Li^+^ between the MoS_2_ layers^4^,^5^,^6^,^7^,^8^,^9^. Although the capacity of MoS_2_ has been greatly improved (theoretical capacity =670 mAh g^−1^), large volume changes occur during charge-discharge cycling, which results in poor cycling stability. Several methods have been reported to successfully improve the cycling stability, such as exfoliation and restacking of MoS_2_ layers^10^, introducing polymers between the MoS_2_ layers,^11^,^12^ and addition of graphene sheets to form composites^13^,^14^. On the other hand, there have been only a few reports on the Na-ion storage of MoS_2_^15^,^16^,^17^,^18^,^19^,^20^,^21^,^22^, which involves the intercalation of 1 Na^+^ per MoS_2_. Considering the advantages of graphene as a highly conductive and stable material, Wang *et al.*^18^ reported MoS_2_—graphene nanocomposite with an improved electrochemical performance when compared to pristine MoS_2_, however, there is no clear evidence of well-ordered stacking of the MoS_2_ and graphene nanosheets among themselves. Three-dimensional (3D) hierarchical micro-spherical architectures with nanostructures as building blocks are considered to be electrochemically and structurally stable morphology that could lead to improved practical application of such active materials in battery systems^21^. Such microspheres exhibit high effective contact areas between active material and the electrolytes, leads to enhanced electrochemical performance (cyclic stability and rate performance) along the sides of short ionic diffusion pathways and resist volume changes due to ‘nanosheets’ as sub-units^23^.

In this work, we are reporting structurally and electrochemically optimized MoS_2_-graphene composites with a unique micro-spherical morphology synthesized via the spray-drying technique, which is an industrial-scale synthesis procedure for a large-scale production of composite powders with a controllable narrow particle size distribution and nano/micro-spherical morphology^24^. We found that the well-ordered and highly miscible hierarchical stacking of graphene-like MoS_2_ and graphene nanosheets could be an efficient structure to enhance the interfacial effect between graphene-like MoS_2_ and graphene, thus taking full advantages from both MoS_2_ and graphene components and achieving high capacity, excellent cycle life and high rate capability as electrode materials for LIBs and SIBs.

## Results and Discussion

**Figure 1:**
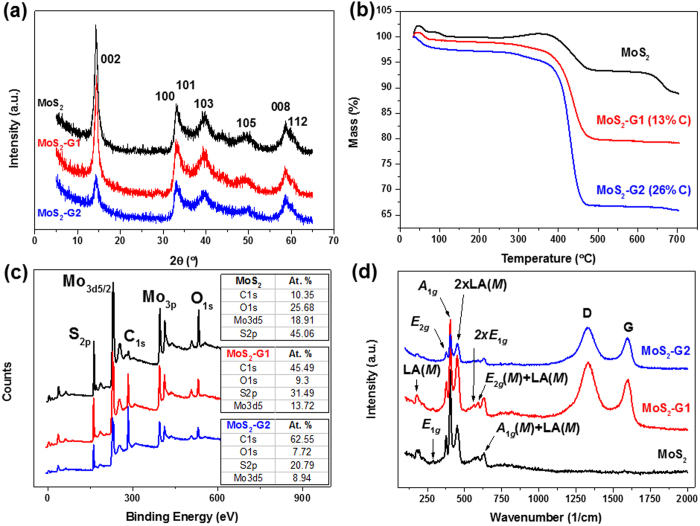
(**a**) XRD patterns, (**b**) TGA curves, (**c**) XPS survey scans, with the insets showing the atomic percentages of the elements in the samples, and (**d**) Raman spectra for the MoS_2_, MoS_2_-G1 and MoS_2_-G2 samples.

After preparation of composite material with various proportions of MoS_2_ and graphene oxide, two proportions of MoS_2_: graphene oxide i.e., 80:20 and 60:40 were optimized based on structural and electrochemical aspects. The synthesis of the MoS_2_-graphene composites with two different graphene ratios is briefly described in Fig. S1 in the Supporting Information. Solutions containing exfoliated MoS_2_ and graphene oxide were mixed in 80:20 (MoS_2_-G1) and 60:40 (MoS_2_-G2) ratios to form a homogenous mixture. The graphene oxide sheets and MoS_2_ sheets were well dispersed among each other and no aggregation were observed after leaving the solution undisturbed overnight. The solutions for the samples were then pumped through a nozzle using a peristaltic pump into a custom-made spray-drying reactor at 350 °C. The resultant black fluffy powders were collected using a cyclone collector attached to the spray drying reactor. The products were then annealed at 800 °C in H_2_/Ar gas flow for 2 h to fully reduce the graphene oxide. The samples were then characterized using X-ray diffraction (XRD) to determine the phase of the material as shown in Fig. 1(a). All of the three samples, MoS_2_, MoS_2_-G1, and MoS_2_-G2, show peaks which can be indexed to hexagonal MoS_2_ (ICDD# 37-1492) and no impurity peaks can be observed. In order to determine the amount of graphene present in the samples after annealing, thermo-gravimetric analysis (TGA) was performed on the samples. The samples were loaded into alumina crucibles and heated in flowing air at the rate of 5 °C/min up to 700 °C. Based on the assumption that all the MoS_2_ is converted into MoO_3_ at 700 °C, the carbon content estimated for MoS_2_-G1 and MoS_2_-G2 is 13 wt% and 26 wt%, respectively, as shown in Fig. 1(b). The composites were also characterized using X-ray photoelectron spectroscopy (XPS) to determine the elemental compositions. Figure 1(c) shows the survey scans of the three samples, and the inset tables indicate the atomic percentages of the elements present in the sample. It should be noted that the samples were subjected to surface etching using ion beams before the XPS characterization. No significant impurity elements were detected from the scans of any of the three samples. The carbon detected on the bare MoS_2_ sample can be attributed to adsorbed CO_2_ on the surface of the samples. The ratio of C to O is roughly 1:2. The atomic percentages (%) of C in MoS_2_-G1 and MoS_2_-G2 agree well with the results from TGA, where the later has a higher amount of graphene sheets present. For all three samples, the ratio of Mo to S is roughly 1:2, which suggest there is minimal or no oxidation of MoS_2_. This is further confirmed by Raman spectroscopy, as shown in Fig. 1(d). All the peaks observed in the Raman spectra below 1000 cm^−1^ can be attributed to hexagonal phase MoS_2_, which is in well agreement with the literature elsewhere^25^. On the other hand, the D and G bands of carbon at 1331 cm^−1^ and 1597 cm^−1^, respectively, can be observed on the spectra of MoS_2_-G1 and MoS_2_-G2.

**Figure 2:**
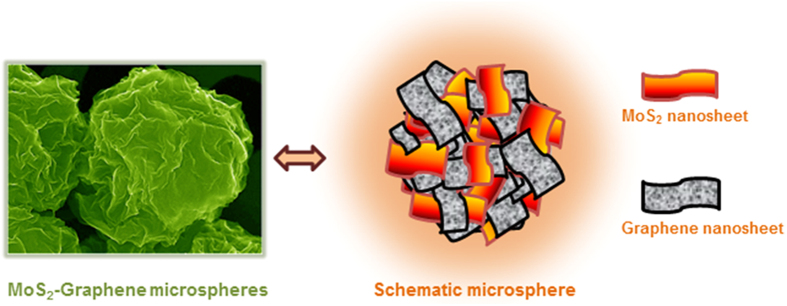
Schematic illustration of MoS_2_-Graphene composite microsphere with hierarchical assembly of MoS_2_ and graphene nanosheets.

**Figure 3:**
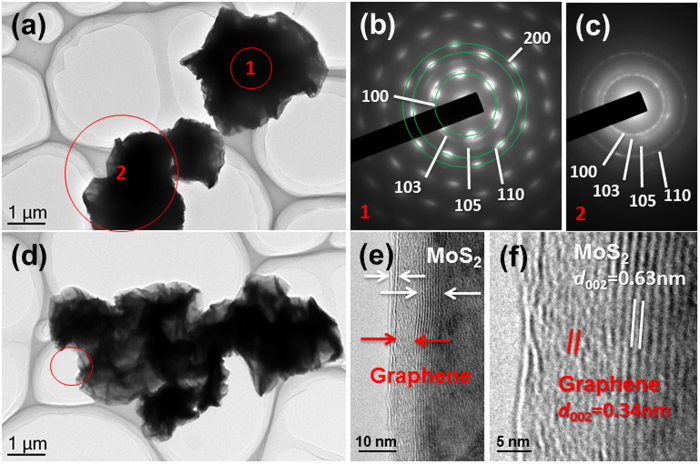
(**a**) TEM image of MoS_2_-G2 microspheres, (**b**, **c**) selected area diffraction (SAED) patterns of corresponding regions marked 1 and 2, respectively, with the patterns indexed to the hexagonal phase, (**d**) TEM image of MoS_2_-G2 sample, (**e**) HRTEM image of marked region in (**d**), and (**f**) magnified image of region from (**e**), revealing the lattice *d*-spacing values of MoS_2_ (0.63 nm) and graphene (0.34 nm).

The morphology of the MoS_2_, MoS_2_-G1 and MoS_2_-G2 samples was investigated using field emission scanning electron microscopy (FESEM), and the images are shown in Fig. S2. All three samples have identical morphology. At low magnification, spheroidal particles with diameters ranging from 1–3 micrometers can be observed. Upon closer inspection at higher magnification, the spheres are found to be made of crumpled sheets, and the kinks can be clearly observed. The same is represented in schematic representation as shown in Fig. 2. The samples were further investigated using transmission electron microscopy (TEM), and the images for MoS_2_-G2 are shown in Fig. 3. TEM analysis shows similar sphere-like morphology. Selected area electron diffraction (SAED) analysis of the sample was performed on a small area inside the sphere and a large area covering the whole sphere. The SAED patterns shown in Fig. 3(b,c) correspond to the areas marked 1 and 2, respectively in Fig. 3(a). The SAED pattern of the area marked 1 (Fig. 3(b)) shows diffuse bright dots due to the single crystalline nature of the MoS_2_ nanosheets. In addition, the spherical morphology of the sample enables the sheets to naturally stack on top of each other rather than in a random stacking. When the SAED pattern was collected from a larger area covering the sides of the sphere, rings were observed instead of the bright dots. Both of the SAED patterns can be indexed to the hexagonal MoS_2_ phase consistent with the XRD results. High resolution TEM (HRTEM) was also used to study the distribution of the MoS_2_ and graphene nanosheets. Figure 3(e) corresponds to the area marked by a red circle in Fig. 3(d). From the image, it can be observed that the graphene nanosheets and MoS_2_ nanosheets are stacked on top of each other, forming a sandwich-like structure. Such a unique spherical microstructure with inter-stacked graphene and MoS_2_ nanosheets is due to a combination of several factors. The miscibility and stability of the graphene oxide and the MoS_2_ nanosheets in aqueous solution is very important to provide the sandwich-like stacking, while the spray-drying process is crucial in providing the spherical morphology of the end product. The thickness of the stacks of graphene and MoS_2_ nanosheets was determined to be from 3–15 layers by studying 10 random areas using HRTEM. Figure 3(f) is an enlarged image of Fig. 3(e), showing the *d*-spacing of the MoS_2_ and graphene nanosheets, which were measured to be 0.63 nm and 0.34 nm, respectively. TEM analysis of MoS_2_ and MoS_2_-G1 was also carried out, yielding similar results. The images and diffraction patterns are presented in Fig. S3 and S4. To further confirm the well-ordered distribution of graphene (carbon), EDX elemental mapping was performed for MoS_2_, MoS_2_-G1 and MoS_2_-G2 samples and represented in Figs S5-S7, respectively and corresponding elemental compositions were tabulated in Tables S1-S3.

**Figure 4:**
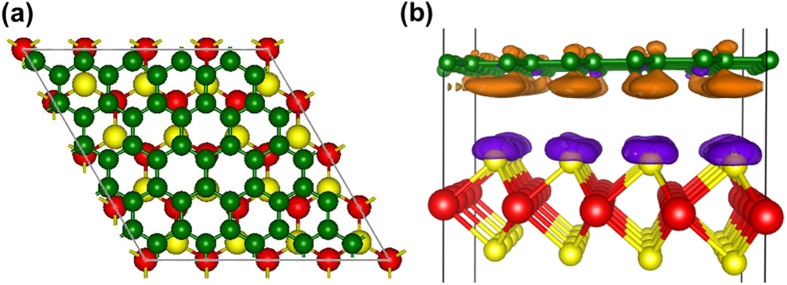
(**a**) Top view of the optimized graphene-MoS_2_ interface, and (**b**) a side view of the three-dimensional charge density difference plot for the interface between a graphene sheet and a MoS_2_ monolayer. Red, yellow, and green balls represent Mo, S, and C atoms, respectively. Purple and orange isosurfaces represent charge accumulation and depletion in the 3D space with an isovalue of 0.001 e/Ǻ^3^.

Computational studies were performed to further understand the interfacial behaviour of MoS_2_ and graphene. A (5 × 5) single graphene layer containing 50 carbon atoms was used to match a (4 × 4) MoS_2_ monolayer containing 16 Mo and 32 S atoms. The lattice mismatch between the graphene and the MoS_2_ monolayer is only 1.3%. Plane-wave basis VASP code was used to perform all the calculations^26^,^27^, implementing the Perdew-Burke-Ernzerhof (PBE) exchange correlation functional^28^. A damped van der Waals correction is also incorporated, based on Grimme’s scheme^29^, to better describe the non-bonding interaction between the graphene and the MoS2 monolayer. In an all-electron description, the projector augmented wave method is used to describe the electron-ion interaction^30^,^31^. The cut-off energy for plane waves was chosen to be 500 eV and the vacuum space is at least 18 Å, which is large enough to avoid the interaction between periodical images. A Monkhorst pack mesh of k-points (3 × 3 × 1) and (5 × 5 × 1) is used respectively to sample the two-dimensional Brillouin zone for geometry optimization and for calculating the charge density. The convergence of the tolerance force on each atom during structure relaxation was set to 0.005 eV/Å. Figure 4(a) presents a top view of the fully relaxed graphene-MoS_2_ geometry. The equilibrium distance between the graphene layer and the top of the MoS_2_ monolayer is calculated to be 3.34 Ǻ. The interface adhesion energy, *E*_*ad*_, was obtained according to the following equation,




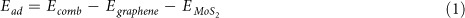




Where *E*_*comb*_*, E*_*graphene*_, and *E*_*MoS2*_ represent the total energy of the relaxed hybrid graphene-MoS_2_ complex, the pure graphene sheet, and the MoS2 monolayer, respectively. The interface binding energy is as high as −2.02 eV for the whole model interface, which indicates very high stability. To characterize the electron coupling at the graphene-MoS_2_ interface, three-dimensional (3D) charge density difference plots were calculated by subtracting the electronic charge of the hybrid graphene-MoS_2_ nanocomposite from those of the separate graphene layer and the MoS_2_ monolayer, as shown in Fig. 4(b). Clearly, there is significant charge transfer from the graphene layer to the top of MoS_2_ surface in the ground electronic state.

**Figure 5:**
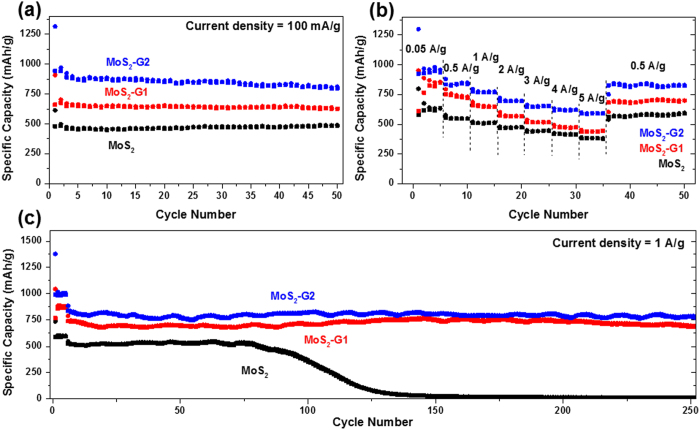
(**a**) Cycling performances in lithium half-cells of all the samples for 50 cycles at 100 mA g^−1^; (**b**) rate capability of all the samples from 0.05 to 5 A g^−1^; and (**c**) cycling performances of all samples for 250 cycles at 1 A g^−1^. The voltage range is 0.01–3 V.

The samples were studied for their lithium storage properties and the results are plotted in Fig. 5 and Fig. S8. All three samples were first cycled at the low current density of 0.1 A g^−1^ over 50 cycles. In the first discharge, MoS_2_-G2 shows the highest capacity at 1300 mAh g^−1^, while MoS_2_-G1 and MoS_2_ show 800 mAh g^−1^ and 630 mAh g^−1^, respectively. Large irreversible capacity is observed for all three samples, as the first charge capacities are 945, 660, and 480 mAh g^−1^ for MoS_2_-G2, MoS_2_-G1, and MoS_2_, respectively as shown in Fig. S10(a). This irreversible capacity can be ascribed to the formation of a solid electrolyte interphase (SEI) layer, which is widely known to occur below 1 V. All three samples showed stable cycling behavior for 50 cycles. The capacities are 800, 630, and 470 mAh g^−1^ for MoS_2_-G2, MoS_2_-G1, and MoS_2_, respectively, at the end of cycling. The samples were further tested for their rate performances up to the current density of 5 A g^−1^. The MoS_2_-G2 sample showed the best rate performance, retaining 590 mAh g^−1^ at 5 A g^−1^. The MoS_2_-G1 and MoS_2_ managed to retain 435 and 387 mAh g^−1^, respectively, at 5 A g^−1^. When the rate was recovered to 0.5 A g^−1^, all the samples showed capacity recovery, where MoS_2_-G2, MoS_2_-G1, and MoS_2_, recovered 820, 680, and 560 mAh g^−1^, respectively. It should be noted that the rate performances improved with increasing graphene content in the samples. This can be justified by the increased conductivity provided by the graphene nanosheets in the samples. In order to investigate the long-term cycling stability, the samples were tested at 50 mA g^−1^ for the initial 5 cycles, then at 1 A g^−1^ up to 250 cycles. The MoS_2_ samples recorded a stable capacity of 500 mAh g^−1^ up to the 75^th^ cycle, and then the capacity gradually faded over 50 cycles. The capacity after the 125^th^ cycle is negligible. This could be attributed to huge volume expansions of pristine MoS_2_ during charge-discharge process and gradually results in pulverization of electrodes over cycle life. The spherical shape of the MoS_2_ sample yielded improved cycling stability and rate performances when compared to the bulk MoS_2_, which has been reported by us previously^15^. Both the MoS_2_-G2, and MoS_2_-G1 samples exhibit stable cycling over 250 cycles, retaining 780 and 700 mAh g^−1^, respectively. From the cycling tests in lithium half-cells, it can be noted that graphene nanosheets in the samples are crucial for improving both the capacity and the cycling performance.

**Figure 6:**
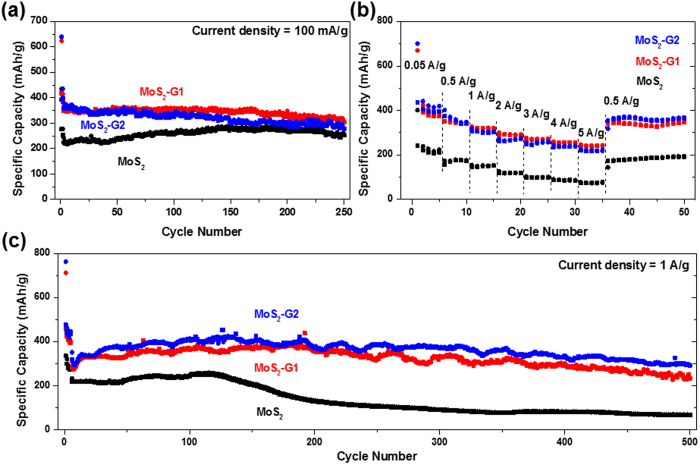
(**a**) Cycling performances in sodium half-cells of all the samples for 50 cycles at 100 mA g^−1^; (**b**) rate capability of all the samples from 0.05 to 5 A g^−1^; and (**c**) cycling performances of all samples for 500 cycles at 1 A g^−1^. The voltage range is 0.01–3 V.

Furthermore, the samples were studied for their sodium storage properties in room-temperature sodium half-cells using similar testing conditions to those for lithium cells (see Fig. 6 and Fig. S9). In the first discharge process (Fig. S9), three voltage plateaus are observed at around ~0.95 V, ~0.65 V and ~0.25 V, which are corresponding to the formation of intermediate Na_*x*_MoS_2_, remaining Na_*1-x*_ ions reacting with MoS_2_ and reduction of Mo^4+^ to metallic Mo along with formation of Na_2_S particulates, respectively. These observations are in consistent with the reports elsewhere^16^,^17^,^18^. However, successive discharge profiles show sloping curves instead of plateaus, which represents the phenomenon of conversion reaction. The same reaction mechanism is expected to happen with Li as standard electrode potential difference (~−0.3 V) from the plateau voltages of initial charge curves (compare Fig. S8 and S9)^16^. Figure. 6 represents cyclic profile, at 0.1 A g^−1^, all three samples show stable cycling behavior over 50 cycles. The initial discharge and charge capacities are 640 and 400, 620 and 420, and 430 and 280 mAh g^−1^, for MoS_2_-G2, MoS_2_-G1, and MoS_2_, respectively. The irreversible capacities of about 35% (see Fig. S10(b)) can be due to the formation of SEI layers. After 50 cycles, both MoS_2_-G2 and MoS_2_-G1 recorded 340 mAh g^−1^, while the MoS_2_ sample recorded 240 mAh g^−1^. The sodium storage capacities of all samples are significantly lower compared to their lithium storage, because only 1 Na^+^ is reacted per MoS_2_, based on the capacity of MoS_2_-G2. This could be due to the sluggish kinetics of the Na^+^ reaction with MoS_2_. Moreover, in contrast to the lithium cells, the increased amount of graphene sheets in MoS_2_-G2 did not yield any increment in capacity when compared to MoS_2_-G1. Then, the samples were also tested for their rate performances, from 0.05 A g^−1^ to 5 A g^−1^. Both MoS_2_-G2 and MoS_2_-G1 showed similar performances, retaining about 230 mAh g^−1^ at 5 A g^−1^, while the MoS_2_ sample retained 74 mAh g^−1^. Although the capacity for both MoS_2_-G2 and MoS_2_-G1 at 5 A g^−1^ is only 230 mAh g^−1^, this result is significantly better compared to other Na-ion battery anode materials^18^,^19^. The long-term cycling stability of the three samples was also tested, where the samples were cycled at 50 mA g^−1^ for the initial 5 cycles and then at 1 A g^−1^ for up to 500 cycles. The MoS_2_ sample showed a slightly consistent capacity up to the 125^th^ cycle, recording 240 mAh g^−1^, and then the capacity gradually decreased to 128 mAh g^−1^ at the 200^th^ cycle. The capacity further decreased to 70 mAh g^−1^ at the 500^th^ cycle. The MoS_2_-G1 sample showed capacity of 375 mAh g^−1^ at the 120^th^ cycle, then a gradual decrease in capacity was observed up to the 500^th^ cycle, where 251 mAh g^−1^ was retained. The MoS_2_-G2 sample showed slightly better performance compared to MoS_2_-G1, recording the capacity of 420 mAh g^−1^ at the 130^th^ cycle and then gradually decreased to 300 mAh g^−1^ at the 500^th^ cycle. The cycling stability is impressive considering that the capacity retention after 500 cycles is 93% of the capacity at the 6^th^ cycle (320 mAh g^−1^). These excellent electrochemical properties in both LIBs and SIBs could be attributed to various reasons such as: (i) the well-ordered stacking and excellent miscibility of the MoS_2_ layers and graphene sheets, which leads to improved conductivity, and thereby, improved cycling performance and rate capability; (ii) suppression of volume changes by the structurally stable nanosheets in microspheres during cycling; and (iii) good penetration of electrolyte into and among the MoS_2_ and graphene nanosheets. In addition, these microspheres with hierarchical nanostructures benefit from both micro-materials (high tap density) and nanomaterials (short Li/Na diffusion pathways), leading to densely packed electrodes and improved battery life in practical applications^32^,^33^,^34^,^35^,^36^. Such hierarchical microstructures can lead to a new class of electrode materials that could be potential candidates for LIBs or SIBs with enhanced cycle life.

In summary, a molybdenum disulfide (MoS_2_)-graphene composite with unique hierarchical microsphere morphology was prepared by the spray-drying technique. The composite microspheres consist of well-ordered stacks with MoS_2_ and graphene nanosheets with a high interfacial binding energy (−2.02 eV). Under testing for their lithium and sodium storage properties, MoS_2_-graphene (26 wt.%) microspheres presented excellent cycling stability and rate capability, with initial discharge capacities of 1300 mAh g^−1^ and 640 mAh g^−1^ at 0.1 A g^−1^ in LIBs and SIBs, respectively. Notably, in SIBs at 1 A g^−1^, MoS_2_-G2 showed 93% capacity retention after 500 cycles. These enhanced electrochemical features are attributable to the unique hierarchical composite microspheres with uniform distribution of graphene nanosheets among MoS_2_ layers.

## Additional Information

**How to cite this article**: Kalluri, S. *et al.* Sodium and Lithium Storage Properties of Spray-Dried Molybdenum Disulfide-Graphene Hierarchical Microspheres. *Sci. Rep.* **5**, 11989; doi: 10.1038/srep11989 (2015).

## Supplementary Material

Supplementary Information

## References

[b1] Kim S. W., Seo D. H., Ma X., Ceder G. & Kang K. Electrode Materials for Rechargeable Sodium‐Ion Batteries: Potential Alternatives to Current Lithium‐Ion Batteries. Adv. Energy Mater. 2, 710–721 (2012).

[b2] Stevens D. A. & Dahn J. R. High Capacity Anode Materials for Rechargeable Sodium‐Ion Batteries. J. Electrochem. Soc. 147, 1271–1273 (2000).

[b3] Kim H. *et al.* Sodium Storage Behavior in Natural Graphite using Ether-based Electrolyte Systems. Adv. Funct. Mater. 25, 534–541 (2015).

[b4] Johnson W. & Worrell W. Lithium and Sodium Intercalated Dichalcogenides: Properties and Electrode Applications. Synth. Met. 4, 225–248 (1982).

[b5] Whittingham M. S. Lithium Batteries and Cathode Materials. Chem. Rev. 104, 4271–4302 (2004). 15669156 10.1021/cr020731c

[b6] Li G. *et al.* Facile Synthesis of Hierarchical Hollow MoS_2_ Nanotubes as Anode Materials for High-performance Lithium-Ion Batteries. CrystEngComm 16, 10731–10922 (2014).

[b7] Zhao C. *et al.* Thin MoS_2_ Nanoflakes Encapsulated in Carbon Nanofibers as High-performance Anodes for Lithium-Ion Batteries. ACS Appl. Mater. Interfaces 6, 6392–6398 (2014). 24701987 10.1021/am4058088

[b8] Chang K. & Chen W. Single-layer MoS_2_/graphene Dispersed in Amorphous Carbon: Towards High Electrochemical Performances in Rechargeable Lithium Ion Batteries. J. Mater. Chem. 21, 17175–17184 (2011).

[b9] Shi Y. *et al.* Self-assembly of Hierarchical MoS_X_/CNT Nanocomposites (2 < x < 3): Towards High Performance Anode Materials for Lithium Ion Batteries. Sci. Rep . 3, 2169 (2013). 10.1038/srep02169 PMC3705413 23835645

[b10] Du G. *et al.* Superior Stability and High Capacity of Restacked Molybdenum Disulfide as Anode Material for Lithium Ion Batteries. Chem. Commun 46, 1106–1108 (2010). 10.1039/b920277c 20126728

[b11] Xiao J. *et al.* Exfoliated MoS_2_ nanocomposite as an anode material for lithium ion batteries. Chem. Mater 22, 4522–4524 (2010).

[b12] Tang H. *et al.* Growth of Polypyrrole Ultrathin Films on MoS_2_ Monolayers as High-Performance Supercapacitor Electrodes. Adv. Mater 27, 1117–1123 (2014). 25529000 10.1002/adma.201404622

[b13] Chang K. & Chen W. L-Cysteine-assisted Synthesis of Layered MoS_2_/graphene Composites with Excellent Electrochemical Performances for Lithium Ion Batteries. ACS Nano 5, 4720–4728 (2011). 21574610 10.1021/nn200659w

[b14] Chang K. & Chen W. *In situ* Synthesis of MoS_2_/graphene Nanosheet Composites with Extraordinarily High Electrochemical Performance for Lithium Ion Batteries. Chem. Commun 47, 4252–4254 (2011). 10.1039/c1cc10631g 21380470

[b15] Park J. *et al.* Discharge Mechanism of MoS_2_ for Sodium Ion Battery: Electrochemical Measurements and Characterization. Electrochim. Acta 92, 427–432 (2013).

[b16] Wang Y.-X. *et al.* Reversible Sodium Storage *via* Conversion Reaction of a MoS_2_-C Composite. Chem. Commun 50, 10730–10733 (2014). 10.1039/c4cc00294f 25084289

[b17] David L., Bhandavat R. & Singh G. MoS_2_/Graphene Composite Paper for Sodium-Ion Battery Electrodes. ACS Nano 8, 1759–1770 (2014). 24446875 10.1021/nn406156b

[b18] Wang Y.-X. *et al.* High-Performance Sodium-Ion Batteries and Sodium-Ion Pseudocapacitors Based on MoS_2_/Graphene Composites. Chem. Eur. J 20, 9607–9612 (2014). 24988995 10.1002/chem.201402563

[b19] Ryu W.-H., Jung J.-W., Park K., Kim S.-J. & Kim I.-D. Vine-like MoS_2_ Anode Materials Self-assembled from 1-D Nanofibers for High Capacity Sodium Rechargeable Batteries. Nanoscale 6, 10975–10981 (2014). 24958669 10.1039/c4nr02044h

[b20] Bang G. S. *et al.* Effective Liquid-Phase Exfoliation and Sodium Ion Battery Application of MoS_2_ Nanosheets. ACS Appl. Mater. Interfaces 6, 7084–7089 (2014). 24773226 10.1021/am4060222

[b21] Wang J. *et al.* An Advanced MoS_2_/Carbon Anode for High-Performance Sodium-Ion Batteries. Small 11, 473–481 (2015). 25256131 10.1002/smll.201401521

[b22] Xie X., Ao Z., Su D., Zhang J. & Wang G. MoS_2_/Graphene Composite Anodes with Enhanced Performance for Sodium-Ion Batteries: The Role of the Two-Dimensional Heterointerface. Adv. Funct. Mater 25, 1393–1403 (2015).

[b23] Bai J., Li X., Liu G., Qian Y. & Xiong S. Unusual Formation of ZnCo_2_O_4_ 3D Hierarchical Twin Microspheres as a High-Rate and Ultralong-Life Lithium-Ion Battery Anode Material. Adv. Funct. Mater. 24, 3012–3020 (2014).

[b24] Son M. Y., Kim J. H. & Kang Y. C. Study of Co_3_O_4_ Mesoporous Nanosheets Prepared by a Simple Spray-drying Process and Their Electrochemical Properties as Anode Material for Lithium Secondary Batteries. Electrochim. Acta 116, 44–50 (2014).

[b25] Frey G. L., Tenne R., Matthews M. J., Dresselhaus M. & Dresselhaus G. Raman and Resonance Raman Investigation of MoS_2_ Nanoparticles. Phys. Rev. B 60, 2883–2892 (1999).

[b26] Kresse G. & Furthmuller J. Efficiency of Ab-initio Total Energy Calculations for Metals and Semiconductors Using a Plane-wave Basis Set. Comput. Mater. Sci. 6, 15–50 (1996). 10.1103/physrevb.54.11169 9984901

[b27] Kresse G. & Furthmuller J. Efficient Iterative Schemes for Ab Initio Total-energy Calculations Using a Plane-Wave Basis Set. Phys. Rev. B 54, 11169–11186 (1996). 10.1103/physrevb.54.11169 9984901

[b28] Perdew J. P., Burke K. & Ernzerhof M. Generalized Gradient Approximation Made Simple. Phys. Rev. Lett. 77, 3865–3868 (1996). 10062328 10.1103/PhysRevLett.77.3865

[b29] Grimme S. Semiempirical GGA-Type Density Functional Constructed with a Long-range Dispersion Correction. J. Comput. Chem. 27, 1787–1799 (2006). 16955487 10.1002/jcc.20495

[b30] Blochl P. E. Projector Augmented-wave Method. Phys. Rev. B 50, 17953–17979 (1994). 10.1103/physrevb.50.17953 9976227

[b31] Kresse G. & Joubert D. From Ultrasoft Pseudopotentials to The Projector Augmented-wave Method. Phys. Rev. B 59, 1758–1775 (1999).

[b32] Wang J. *et al.* Accurate Control of Multishelled Co_3_O_4_ Hollow Microspheres as High-Performance Anode Materials in Lithium-Ion Batteries. Angew. Chem. Int. Ed. 52, 6417–6420 (2013). 10.1002/anie.201301622 23649876

[b33] Zhang C., Chen Z., Guo Z. & Lou X. W. Additive-free Synthesis of 3D porous V_2_O_5_ Hierarchical Microspheres with Enhanced Lithium Storage Properties. Energy Environ. Sci. 6, 974–978 (2013).

[b34] Ren H. *et al.* Multishelled TiO_2_ Hollow Microspheres as Anodes with Superior Reversible Capacity for Lithium Ion Batteries. Nano Lett. 14, 6679–6684 (2014). 25317725 10.1021/nl503378a

[b35] Kalluri S., Seng K. H., Guo Z., Liu H. K. & Dou S. X. Electrospun Lithium Metal Oxide Cathode Materials for Lithium-ion Batteries. RSC Adv. 3, 25576–25601 (2013).

[b36] Xu S. *et al.* α-Fe_2_O_3_ Multi-shelled Hollow Microspheres for Lithium Ion Battery Anodes with Superior Capacity and Charge Retention. Energy Environ. Sci. 7, 632–637 (2014).

