# Supplementary material for: Sodium and Lithium Storage Properties of Spray-Dried Molybdenum Disulfide-Graphene Hierarchical Microspheres
Source: Sci Rep. 2015 Jul 15;5:11989. doi: 10.1038/srep11989 (PMC4648418; doi:10.1038/srep11989)
Supplement: Supplementary Information [file srep11989-s1.pdf]

Supporting Information

Sodium and Lithium Storage Properties of Spray-Dried Molybdenum Disulfide-Graphene Hierarchical Microspheres

Sujith Kalluri,[a,b,¥] Kuok Hau Seng,[a,¥] Zaiping Guo,[a,b*] Aijun Du,[c] Konstantin Konstantinov,[a,b*] Hua Kun Liu,[a] Shi Xue Dou[a]

[a]*Institute for Superconducting and Electronic Materials, University of Wollongong, NSW 2500, Australia.*

[b]*School of Mechanical, Materials and Mechatronics Engineering, University of Wollongong, NSW 2500, Australia.*

[c]*School of Chemistry, Physics and Mechanical Engineering, Queensland University of Technology, Brisbane, Queensland 4001, Australia.*

Corresponding Authors' email: zguo@uow.edu.au, konstan@uow.edu.au

[¥]Equally contributed to the first authorship

Experimental Methods

Two ratios of MoS_2-graphene oxide (MoS_2-G1 = 80:20; MoS_2-G2 = 60:40) in suspensions (2 L; 2 mg/mL) were prepared by stirring for 30 min and sonication for 10 min. The synthesis methods for the MoS_2 and graphene oxide suspensions are reported as below. The MoS_2-graphene oxide suspension was spray-dried using a feeding rate of 200 mL h^{-1} and temperature of 350°C. The resultant black fluffy powder was collected using a cyclone collector. MoS_2 samples were also prepared using the same method without the addition of graphene oxide. The spray-dried products were then annealed at 800°C for 2 hours in 5% H_2/95% Ar flow to reduce the graphene oxide.

For electrochemical analysis, the samples were mixed with sodium carboxymethyl cellulose (Sigma Aldrich), polyacrylic acid (Sigma Aldrich), and carbon black (TimCal) in a ratio of 80:5:5:10. De-ionised water was added to form a homogeneous slurry, which was then pasted on copper foil using a doctor blade. The electrodes were dried under vacuum at 150°C for 3 h and then punched into 0.7 cm^2 round disks for electrochemical tests. 2032 type coin cells were assembled in an argon-filled glove box with lithium metal as the counter electrode, microporous polyethylene as the separator, and 1.0 M $LiPF_6$ in ethylene carbonate/dimethyl carbonate/diethyl carbonate + 5 wt% fluoroethylene carbonate (EC/DMC/DEC; 3/4/3; Novolyte) as the electrolyte. For sodium cells, sodium foil was used as the counter electrode, glassy fibers as the separator, and 1 M $NaClO_4$ in propylene carbonate (PC) + 5 wt% fluoroethylene carbonate (FEC) as the electrolyte. FEC is a structural stabilizing electrolyte additive for the solid electrolyte interphase (SEI) film and thereby enhance the ionic intercalation/de-intercalation mechanism.[1,2] The loading amount of active materials for all electrodes was 1.00 ± 0.05 mg cm^{-2}.

Preparation of exfoliated MoS_2 suspension

2 g MoS_2 (Sigma Aldrich) was soaked in 1.5 mol equivalent of n-butyl lithium (1.6 M in hexane; Sigma Aldrich) for at least 8 hours in a vial in an argon glove box. Then, the vial was sealed and removed from the glove box. The mixture was left to settle in a fume cupboard for 30 min, and the brownish liquid on top was decanted, leaving a dark precipitate. Then, the precipitate was transferred into a large beaker containing 500 mL deionized water and left under stirring for 30 min to form a homogeneous black suspension. A vigorous reaction was observed during the addition due to the reaction of the remaining n-butyl lithium with water and the exfoliation of the MoS_2. Then, the black suspension was centrifuged and washed with deionized water 3 times to remove the lithium salts. The black precipitate was then re-dispersed in 400 mL of deionized water and sonicated for 10 min to form a homogeneous suspension. Bulk and un-exfoliated MoS_2 was removed by centrifuging the suspension at 2000 rpm for 10 min. The amount of MoS_2 in the solution (mg/mL) was estimated by drying 50 mL of the suspension at 60 ℃ under vacuum.

Preparation of graphene oxide solution

3 g graphite flakes (Sigma Aldrich) were added to 360 mL sulfuric acid (Sigma Aldrich) and 40 mL phosphoric acid (Sigma Aldrich). 18 g $KMnO_4$ was slowly added under stirring to form a viscous dark greenish mixture. The mixture was then kept in an oil bath at 50 ℃ for 20 h to form a brownish mixture. Then, the mixture was left to cool to room temperature. The mixture was then poured into 400 mL ice and 3 mL of 30% hydrogen peroxide (Sigma Aldrich). The mixture was then washed with 2% HCl for 3 times, ethanol for 3 times, and deionized water for 5 times. The brown graphite oxide precipitate was re-dispersed in 1 L deionized water stirred for 30 min and sonicated for 10 min. The amount of graphene oxide in the solution (mg/mL) was estimated by drying 50 mL of the suspension at 60 ℃ under vacuum.

Table [S1]

MoS_2		
**Elements**	**Series**	**Atomic weight (%)**
Cu	K-series	71.52
Mo	L-series	8.04
S	K-series	20.44
		**Total:** 100.00

Table [S2]

MoS_2-G1		
**Elements**	**Series**	**Atomic weight (%)**
Cu	K-series	50.42
Mo	L-series	4.91
S	K-series	10.94
C	K-series	33.73
		**Total:** 100.00

Table [S3]

MoS_2-G2		
**Elements**	**Series**	**Atomic weight (%)**
Cu	K-series	41.27
Mo	L-series	3.78
S	K-series	8.61
C	K-series	46.34
	**Total:**	100.00

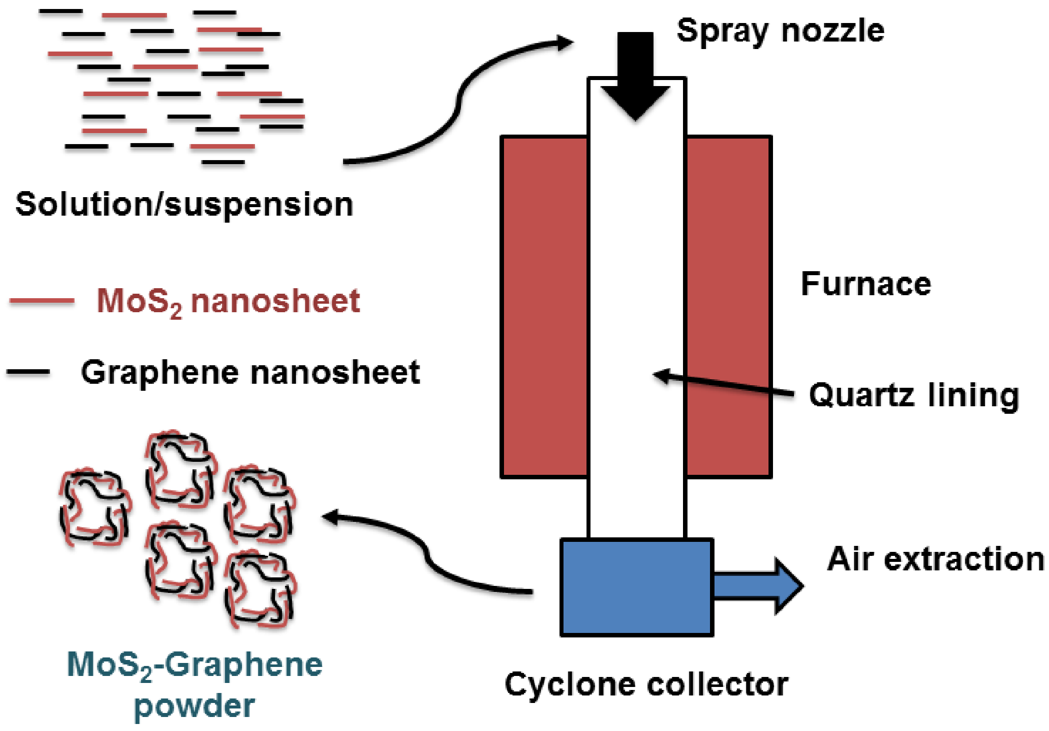

Figure [S1]

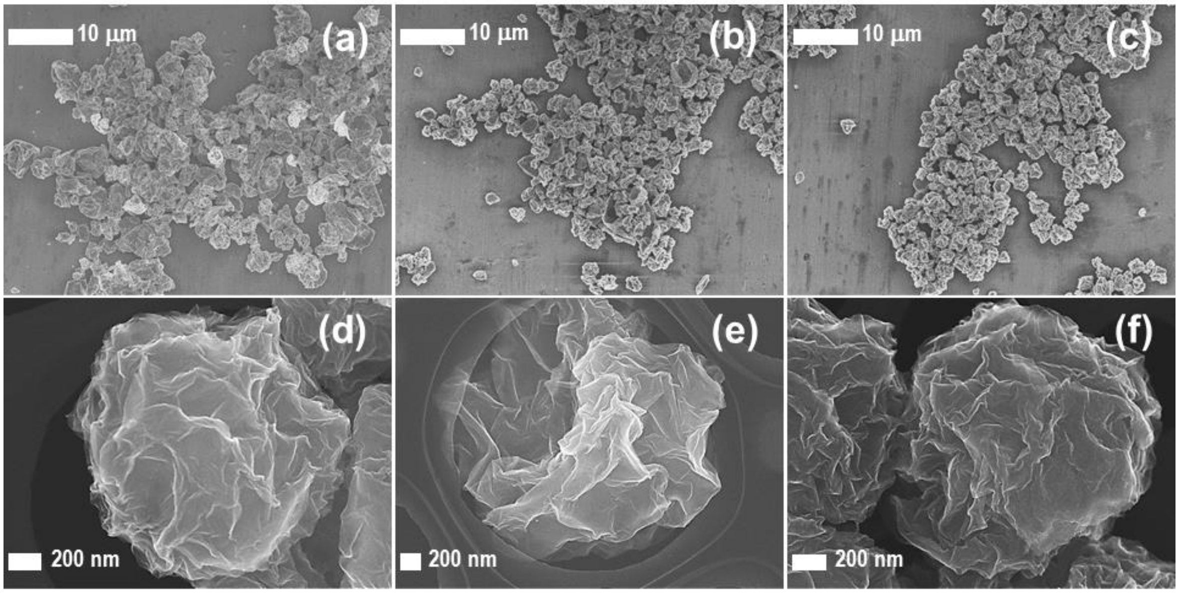

Figure [S2]

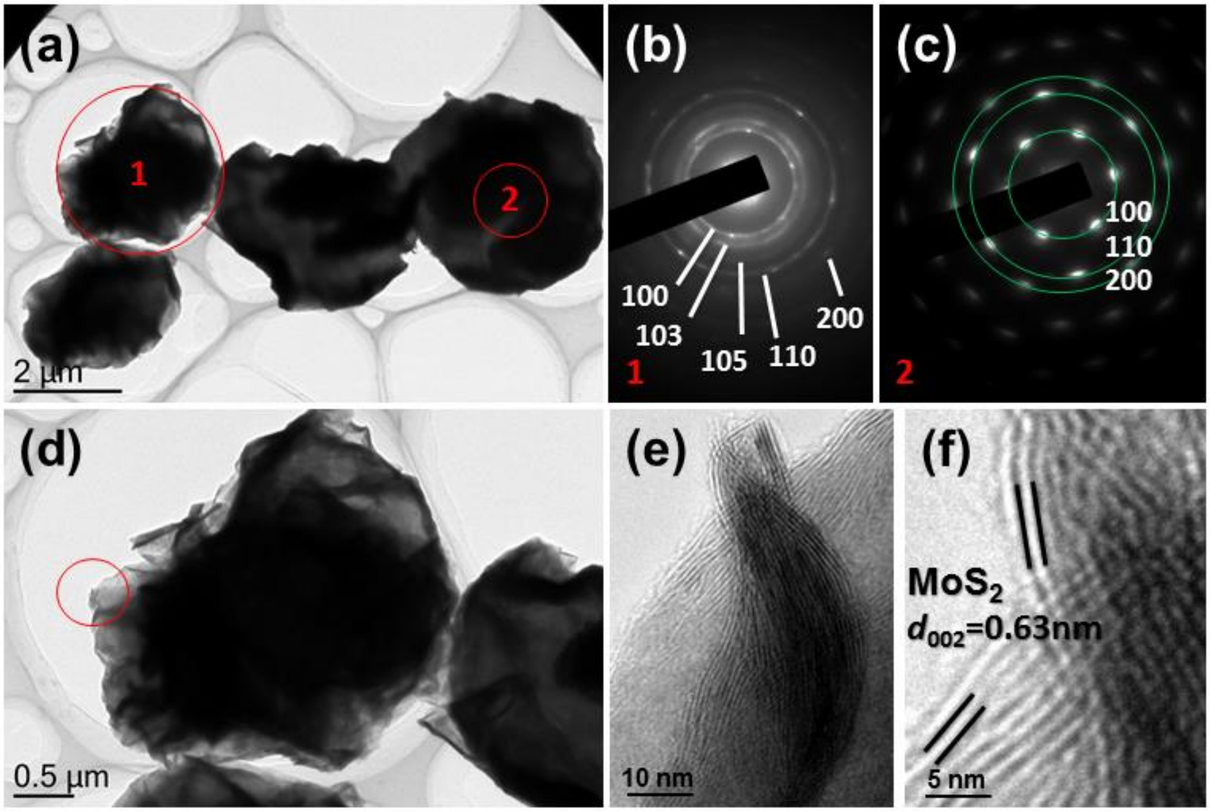
(a)
1
2
2 µm
(b)
100
103
105
110
200
1
(c)
100
110
200
2
(d)
0.5 µm
(e)
10 nm
(f)
MoS$_2$
d_{002}=0.63nm
5 nm

Figure [S3]

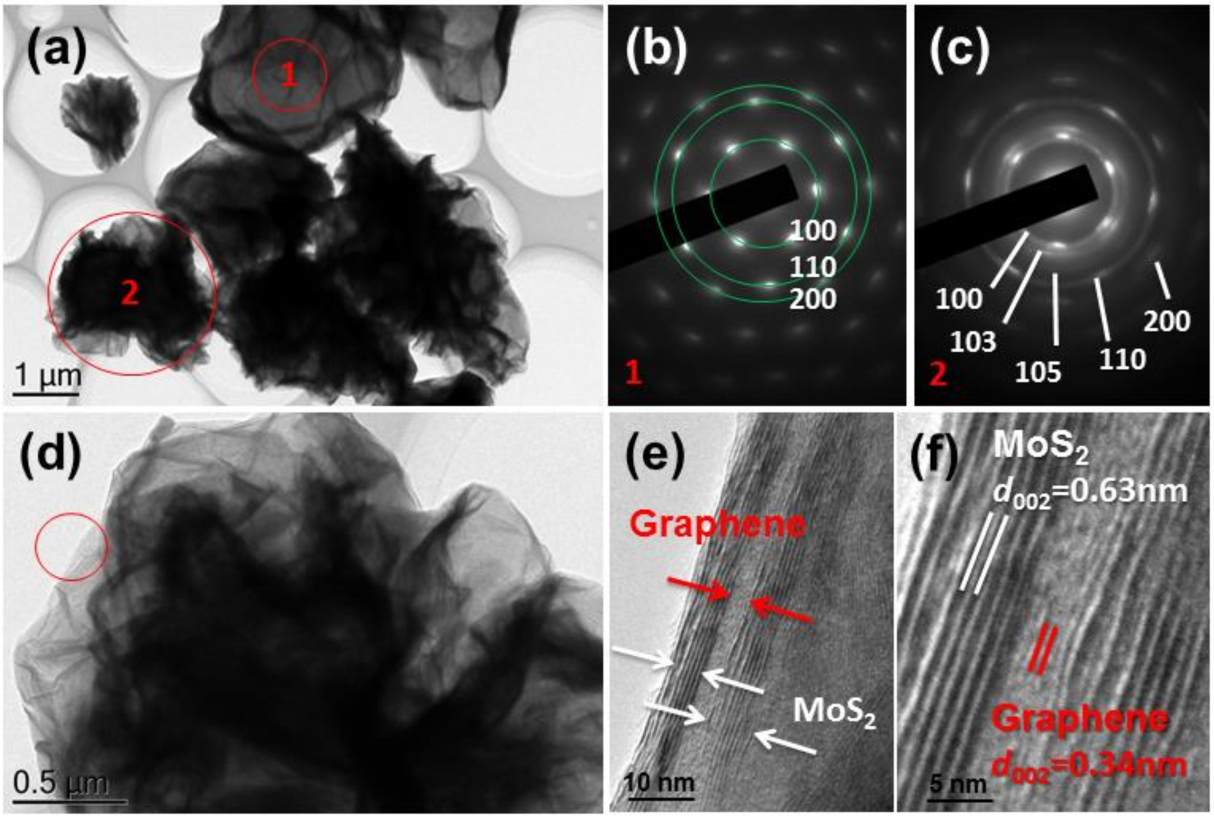

(a)
1
2
1 µm
(b)
100
110
200
1
(c)
100
103
105
110
200
2
(d)
0.5 µm
(e)
Graphene
MoS2
10 nm
(f)
MoS2
d002=0.63nm
Graphene
d002=0.34nm
5 nm

Figure [S4]

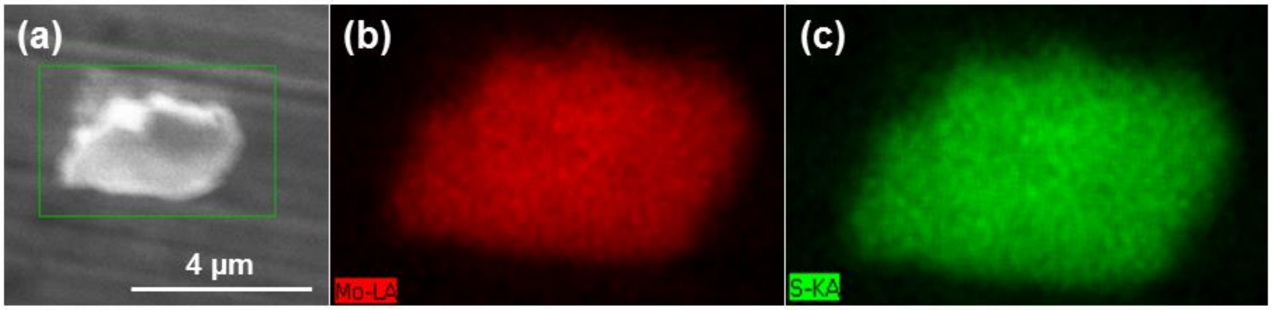

Figure [S5]

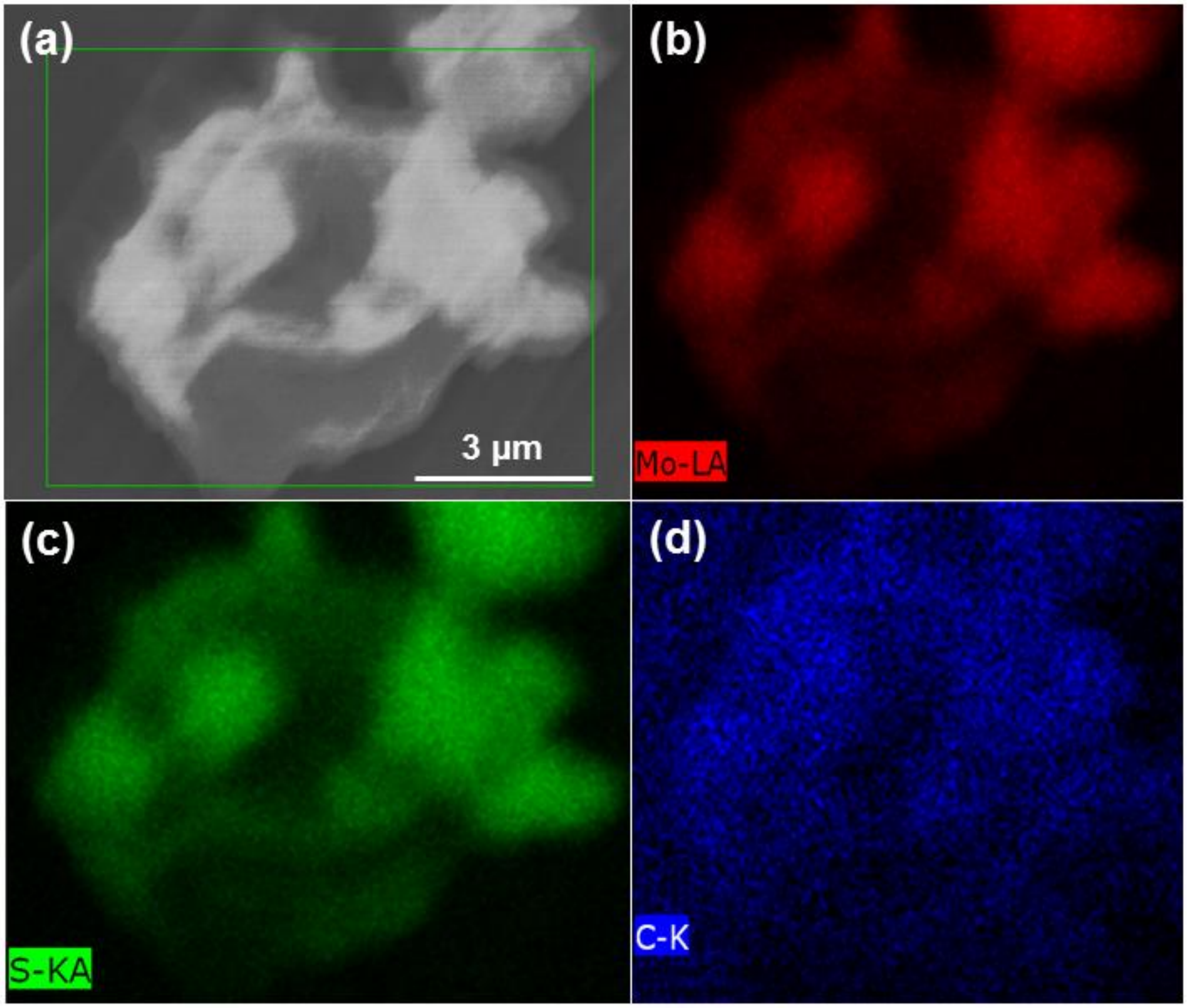

Figure [S6]

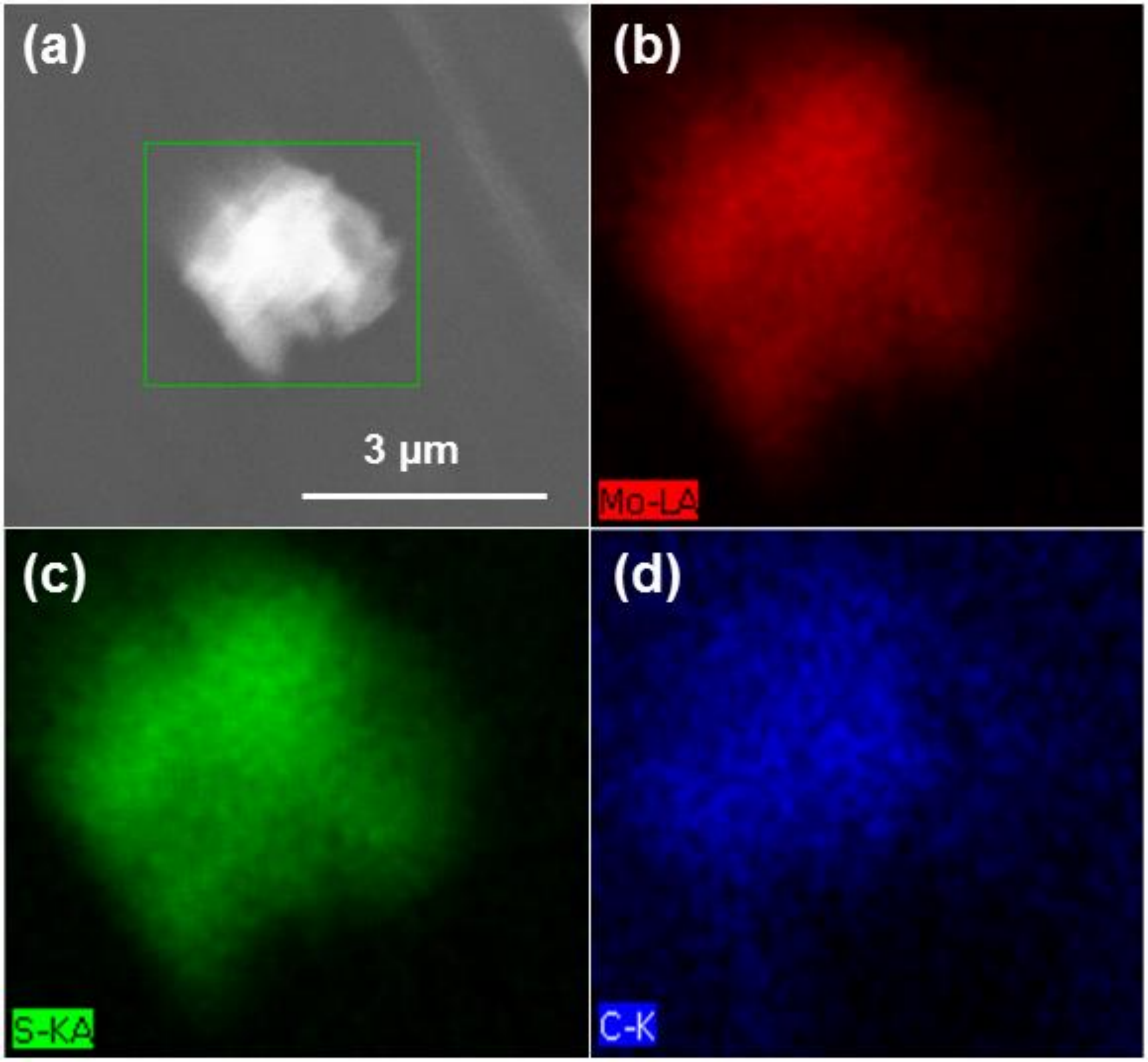

Figure [S7]

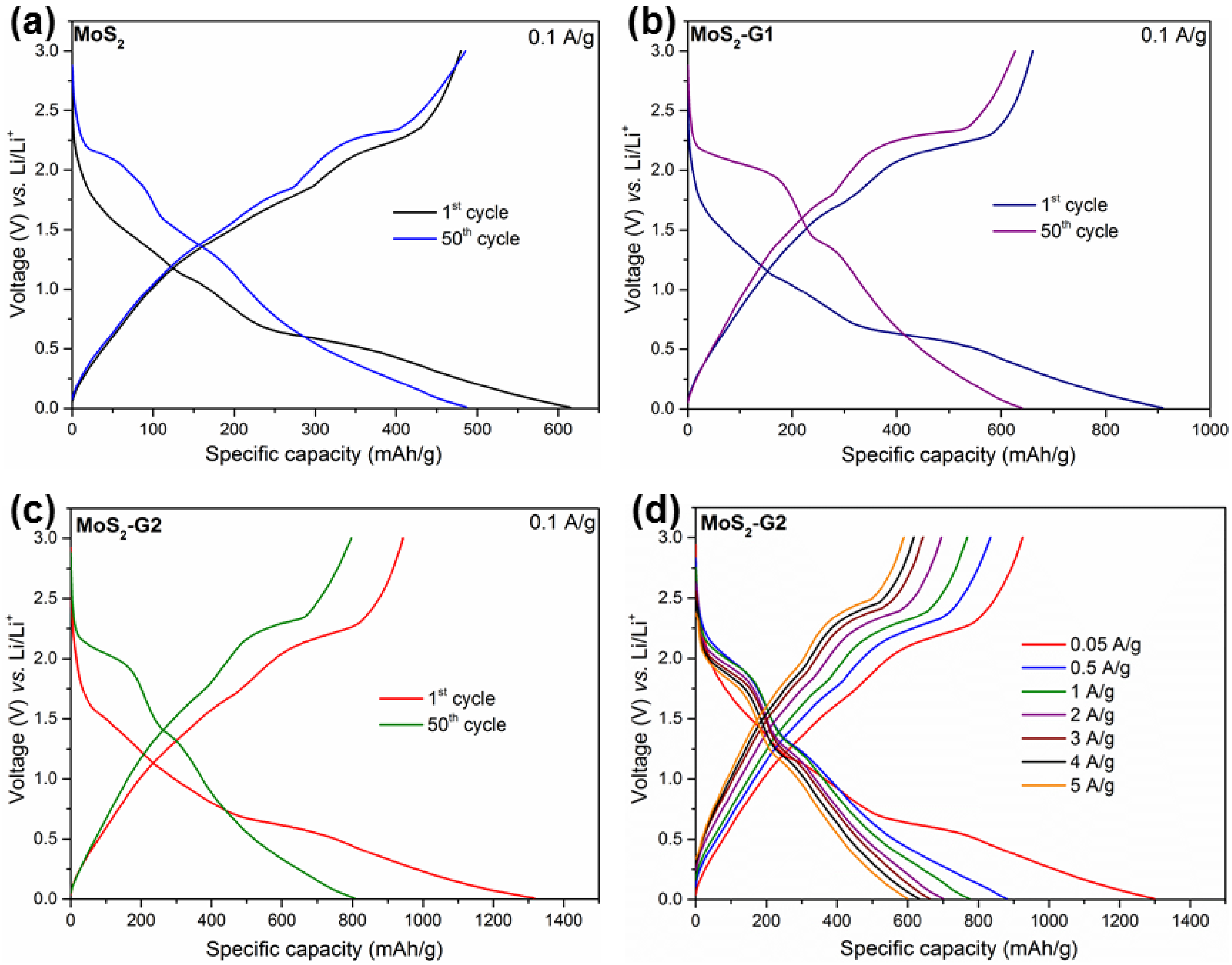
(a)
MoS2
0.1 A/g
1st cycle
50th cycle
Voltage (V) vs. Li/Li+
Specific capacity (mAh/g)
(b)
MoS2-G1
0.1 A/g
1st cycle
50th cycle
Voltage (V) vs. Li/Li+
Specific capacity (mAh/g)
(c)
MoS2-G2
0.1 A/g
1st cycle
50th cycle
Voltage (V) vs. Li/Li+
Specific capacity (mAh/g)
(d)
MoS2-G2
0.05 A/g
0.5 A/g
1 A/g
2 A/g
3 A/g
4 A/g
5 A/g
Voltage (V) vs. Li/Li+
Specific capacity (mAh/g)

Figure [S8]

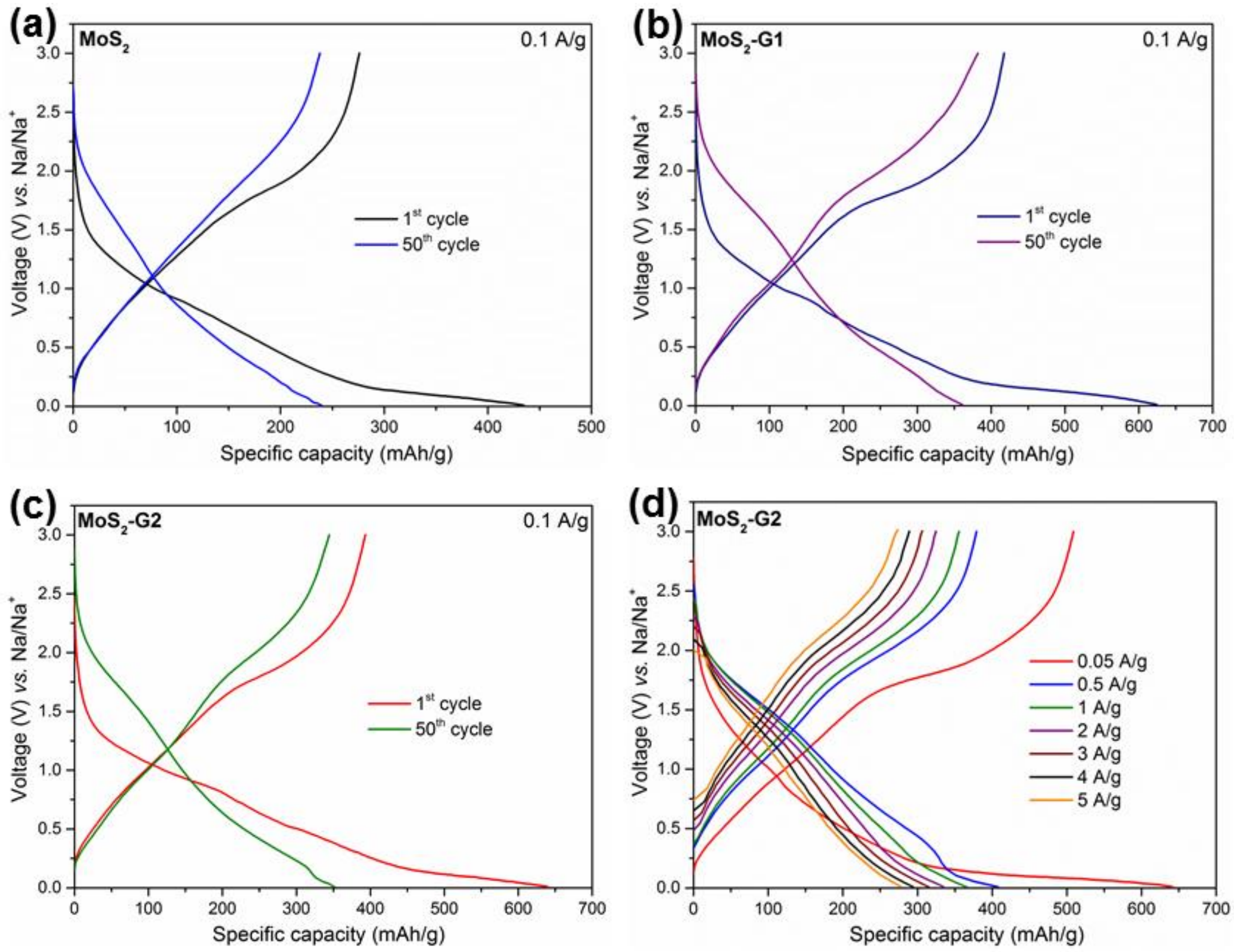

(a)
MoS2
0.1 A/g
1st cycle
50th cycle
Voltage (V) vs. Na/Na+
Specific capacity (mAh/g)
(b)
MoS2-G1
0.1 A/g
1st cycle
50th cycle
Voltage (V) vs. Na/Na+
Specific capacity (mAh/g)
(c)
MoS2-G2
0.1 A/g
1st cycle
50th cycle
Voltage (V) vs. Na/Na+
Specific capacity (mAh/g)
(d)
MoS2-G2
0.05 A/g
0.5 A/g
1 A/g
2 A/g
3 A/g
4 A/g
5 A/g
Voltage (V) vs. Na/Na+
Specific capacity (mAh/g)

Figure [S9]

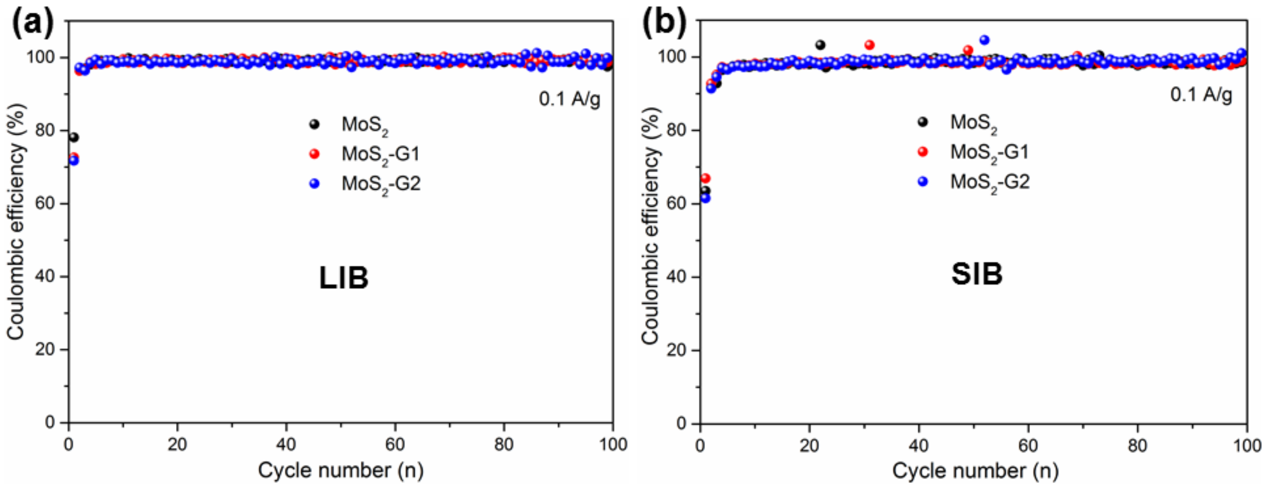

Figure [S10]

References

1. Darwiche, A. *et al*. Better Cycling Performances of Bulk Sb in Na-Ion Batteries Compared to Li-Ion Systems: An Unexpected Electrochemical Mechanism. *J. Am. Chem. Soc.* **134**, 20805-20811 (2012).
2. Etacheri, V. *et al*. Effect of Fluoroethylene Carbonate (FEC) on the Performance and Surface Chemistry of Si-Nanowire Li-Ion Battery Anodes. *Langmuir* **28**, 965-976 (2012).

Table captions

Table [S1] EDX elemental composition of MoS_2 single microsphere (Cu is from substrate used for EDX analysis).

Table [S2] EDX elemental composition of MoS_2-G1 single microsphere (Cu is from substrate used for EDX analysis).

Table [S3] EDX elemental composition of MoS_2-G2 single microsphere (Cu is from substrate used for EDX analysis).

Figure captions

Figure [S1] Schematic illustration of spray-drying method for synthesizing MoS_2-graphene composite microspheres.

Figure [S2] (a-c) SEM images of the MoS_2, MoS_2-G1, and MoS_2-G2 samples, respectively, and (d-f) corresponding magnified SEM images.

Figure [S3] (a) TEM image of MoS_2 microspheres, (b,c) SAED patterns of corresponding regions marked 1 and 2, respectively, with the patterns indexed to the hexagonal phase, (d) TEM image of MoS_2 sample, (e) HRTEM image of marked region in (d), and (f) magnified image of region from (e), revealing the lattice *d*-spacing value of MoS_2 (0.63 nm).

Figure [S4] (a) TEM image of MoS_2-G1 microspheres, (b,c) SAED patterns of corresponding regions marked 1 and 2, respectively, with the patterns indexed to hexagonal phase; (d) TEM image of MoS_2-G1 sample, (e) HRTEM image of marked region in (d), and (f) magnified image of region from (e), revealing the lattice *d*-spacing values of MoS_2 (0.63 nm) and graphene (0.34 nm).

Figure [S5] (a) SEM image of MoS_2 single microsphere, (b, c) EDX elemental mapping corresponding to Mo and S of marked region in (a).

Figure [S6] (a) SEM image of MoS_2-G1 single microsphere, (b-d) EDX elemental mapping corresponding to Mo, S and C of marked region in (a).

Figure [S7] (a) SEM image of MoS_2-G2 single microsphere, (b-d) EDX elemental mapping corresponding to Mo, S and C of marked region in (a).

Figure [S8] Charge-discharge behavior at 1st and 50th cycles of (a) MoS_2, (b) MoS_2-G1, and (c) MoS_2-G2 samples at current density of 0.1 A/g and voltage range 0.01 - 3 V *vs.* Li/Li^+; (d) charge-discharge behavior of MoS_2-G2 sample at different current densities from 0.05 A/g to 5 A/g.

Figure [S9] Charge-discharge behavior at 1st and 50th cycles of (a) MoS_2, (b) MoS_2-G1, and (c) MoS_2-G2 samples at current density of 0.1 A/g and voltage range 0.01 - 3 V *vs.* Na/Na^+; (d) charge-discharge behavior of MoS_2-G2 sample at different current densities from 0.05 A/g to 5 A/g.

Figure [S10] Coulombic efficiency of MoS_2, MoS_2-G1, and MoS_2-G2 samples in (a) LIB and (b) SIB, respectively.
